# A range of C∊3–C∊4 interdomain angles in IgE Fc accommodate binding to its receptor CD23

**DOI:** 10.1107/S2053230X14003355

**Published:** 2014-02-20

**Authors:** Balvinder Dhaliwal, Marie O. Y. Pang, Daopeng Yuan, Andrew J. Beavil, Brian J. Sutton

**Affiliations:** a Randall Division of Cell and Molecular Biophysics, King’s College London, New Hunt’s House, Guy’s Campus, London SE1 1UL, England

**Keywords:** immunoglobin E, CD23, Fc∊3-4

## Abstract

The antibody IgE plays a central role in allergic disease, functioning principally through two cell-surface receptors: Fc∊RI and CD23. Fc∊RI on mast cells and basophils mediates the immediate hypersensitivity response, whilst the interaction of IgE with CD23 on B cells regulates IgE production. Crystal structures of the lectin-like ‘head’ domain of CD23 alone and bound to a subfragment of IgE consisting of the dimer of C∊3 and C∊4 domains (Fc∊3-4) have recently been determined, revealing flexibility in the IgE-binding site of CD23. Here, a new crystal form of the CD23–Fc∊3-4 complex with different molecular-packing constraints is reported, which together with the earlier results demonstrates that conformational variability at the interface extends additionally to the IgE Fc and the quaternary structure of its domains.

## Abstract 2

The structure of a new crystal form of IgE-Fc in complex with its B-cell receptor CD23 has been determined. The structure reveals that there is conformational variability at the interface in both IgE-Fc and CD23.

## 1. Introduction   

The prevalence of allergy is increasing worldwide (Finkelman & Vercelli, 2007 ▶). Allergic diseases, including asthma, hay fever and anaphylactic shock, are commonly triggered by environmental allergens, resulting in an inflammatory response. The antibody immuno­globulin E (IgE) plays a central role in the mechanism of allergic disease (Gould & Sutton, 2008 ▶). IgE binds allergens through its Fab regions, and interacts with two very different cell-surface receptors, Fc∊RI and CD23 (also known as Fc∊RII), *via* its Fc region. Fc∊RI binds IgE with high affinity (*K*_a_ = 10^10^–10^11^ *M* ^−1^) and is responsible for allergic sensitization and the immediate hyper­sensitivity response in which minute amounts of allergen cross-link Fc∊RI-bound IgE on mast cells and basophils, triggering cellular degranulation. The IgE-binding α-chain of Fc∊RI consists of two extracellular Ig-like domains (sFc∊RIα; Garman *et al.*, 2000 ▶; Holdom *et al.*, 2011 ▶).

CD23, expressed on B cells and antigen-presenting cells, belongs to the C-type lectin-like domain (CTLD) superfamily (Zelensky & Gready, 2005 ▶). Some CTLD-containing proteins interact with the carbohydrate moieties of glycoproteins *via* a bound Ca^2+^ ion, whilst others bind neither carbohydrate nor Ca^2+^ and interact with other ligands such as proteins (Natarajan *et al.*, 2002 ▶). In its cell membrane-bound form, CD23 consists of three CTLD heads connected to the membrane by a trimeric α-helical coiled-coil stalk (Beavil *et al.*, 1992 ▶). A single head domain binds to human IgE Fc in a carbohydrate-independent manner with an affinity *K*_a_ of ∼10^6^ *M* ^−1^ (Shi *et al.*, 1997 ▶; Hibbert *et al.*, 2005 ▶; Dhaliwal *et al.*, 2012 ▶). However, CD23 does bind Ca^2+^ (Hibbert *et al.*, 2005 ▶; Wurzburg *et al.*, 2006 ▶), and this has recently been to shown to induce structural changes in CD23 that lead to a 30-­fold increase in the affinity of a single head domain for IgE (Yuan *et al.*, 2013 ▶). Moreover, the avidity of the CD23 trimer further enhances the interaction with IgE immune complexes (Dierks *et al.*, 1993 ▶; McCloskey *et al.*, 2007 ▶).

The stalk region of CD23 is susceptible to attack by endogenous proteases such as ADAM10 (Weskamp *et al.*, 2006 ▶), releasing soluble trimeric and monomeric forms of CD23. The house dust mite allergenic protease Der p 1 generates a soluble monomeric form of CD23 consisting of just the lectin head domain, termed derCD23 (Schulz *et al.*, 1997 ▶). Depending on the oligomerization state of CD23, its interaction with soluble or membrane IgE either up-regulates or down-regulates IgE production (Gould & Sutton, 2008 ▶; McCloskey *et al.*, 2007 ▶; Cooper *et al.*, 2012 ▶). Binding of IgE immune complexes to CD23 also enhances allergen presentation (Carlsson *et al.*, 2007 ▶), the process by which CD23 internalizes allergens and recycles peptides complexed with MHC class II molecules to the cell surface for T-cell recognition. The CD23–IgE interaction is therefore a target for therapeutic intervention in allergic disease.

In a recent study, we determined the crystal structure of derCD23 bound to Fc∊3-4 (a subfragment of IgE-Fc consisting of a dimer of the C∊3 and C∊4 domains; Dhaliwal *et al.*, 2012 ▶). The structure explained the known 2:1 stoichiometry, with one CD23 head domain binding to each heavy chain of IgE, and also revealed that conformational changes in Fc∊3-4 upon binding derCD23 are incompatible with sFc∊RIα binding and *vice versa*. In the present study, we have identified a new crystal form of the derCD23–Fc∊3-4 complex in which different crystal-packing forces constrain the Fc domains. This structure, together with the earlier data, shows that a range of C∊3–C∊4 interdomain angles are compatible with CD23 binding and that this protein–protein interface displays remarkable conformational variability in both partners.

## 2. Materials and methods   

### 2.1. Protein purification   

Recombinant human derCD23 (Ser156–Glu298) was expressed in *E. coli*, refolded and purified as described previously (Hibbert *et al.*, 2005 ▶; Dhaliwal *et al.*, 2012 ▶). Briefly, derCD23 was purified on a heparin Sepharose column (GE Healthcare) pre-equilibrated with 25 m*M* Tris–HCl pH 7.5 (purification buffer) and eluted with 25 m*M* Tris–HCl pH 7.5, 200 m*M* NaCl. Pooled fractions were concentrated to 1 ml and loaded onto a HiLoad 16/60 Superdex G75 column (GE Healthcare) pre-equilibrated and subsequently washed with purification buffer. The correct folding of derCD23 was assessed by one-dimensional ^1^H-NMR at 500 MHz (large dispersion and strong signals of methyl groups were observed between 1.0 and −1.0 p.p.m.).

The recombinant human Fc∊3-4 gene (Cys328–Lys547, with N-­terminal sequence Ala-Asp-Pro) was synthesized, cloned and transiently transfected into HEK293 cells as described previously (Dhaliwal *et al.*, 2012 ▶). The supernatants were harvested 6 d after transfection. Human Fc∊3-4 was then purified by cation-exchange chromatography on an SPHP matrix (GE Healthcare) in 50 m*M* sodium acetate buffer pH 6.0, followed by gel filtration on a Superdex S200 matrix (GE Healthcare) in PBS pH 7.4.

### 2.2. Crystallization and data collection   

Prior to crystallization, Fc∊3-4 was concentrated to 13 mg ml^−1^, and derCD23 to 11.5 mg ml^−1^, in 25 m*M* Tris–HCl pH 7.5, 20 m*M* NaCl, 0.05%(*w*/*v*) sodium azide (storage buffer). derCD23–Fc∊3-4 complex crystals were obtained by the sitting-drop vapour-diffusion method. Crystals were grown by mixing 150 nl of a protein solution consisting of 0.2 m*M* derCD23 (3.2 mg ml^−1^), 0.1 m*M* Fc∊3–4 (5 mg ml^−1^) with 75 nl reservoir solution consisting of 100 µl 22–­26%(*w*/*v*) PEG 1500, 20%(*v*/*v*) glycerol. The largest crystals, which grew within a week at 291 K in the absence of any added CaCl_2_, were ∼500 µm in length and were obtained by micro-seeding. Several small plate-like crystals were added to 50 µl reservoir solution, vortexed vigorously for 2 min, diluted 1000-fold with storage buffer and added to the protein solution at a tenth of the final volume before setting up crystallization optimization trials.

Diffraction data were collected at 100 K on beamline I04 of the Diamond Light Source synchrotron, Harwell, England. No additional cryoprotectant solution was required.

### 2.3. Structure determination   

Indexing and integration of the data were carried out using *MOSFLM* (Powell, 1999 ▶; Battye *et al.*, 2011 ▶) and merging was carried out with *SCALA* (Evans, 2006 ▶). The derCD23–Fc∊3-4 complex structure was solved by molecular replacement with *Phaser* (McCoy *et al.*, 2007 ▶) using the previously determined derCD23–Fc∊3-4 crystal structure (PDB entry 4ezm; Dhaliwal *et al.*, 2012 ▶) as the search model.

Owing to the high degree of noncrystallographic symmetry (NCS), reflections were selected for the *R*_free_ set in thin resolution shells (Fabiola *et al.*, 2006 ▶). Iterative cycles of refinement using *PHENIX* (Adams *et al.*, 2011 ▶), *REFMAC*5 (Murshudov *et al.*, 2011 ▶) and *BUSTER-TNT* (Smart *et al.*, 2012 ▶) were alternated with manual model building with *Coot* (Emsley *et al.*, 2010 ▶). The model was built into 2*F*_o_ − *F*_c_ composite omit 2*F*_o_ − *F*_c_ and *F*_o_ − *F*_c_ electron-density maps in order to minimize model bias. Carbohydrate atoms were subsequently added to the structure. Tight NCS restraints were used initially, and were gradually relaxed with local structure similarity restraints (or ‘local NCS’; Murshudov *et al.*, 2011 ▶; Smart *et al.*, 2012 ▶) applied. TLS groups (Painter & Merritt, 2005 ▶) were generated using the *TLSMD* web server (Painter & Merritt, 2006 ▶). Carbohydrate atoms were subsequently built into the structure. Data-processing and refinement statistics are shown in Table 1 ▶. The final coordinates and structure factors have been deposited in the Protein Data Bank under accession code 4ki1.

*Hingefind* (Wriggers & Schulten, 1997 ▶), *DynDom* (Hayward & Berendsen, 1998 ▶), *CONTACT* (Winn *et al.*, 2011 ▶) and *PISA* (Krissinel & Henrick, 2007 ▶) were used for structural analysis. All of the structural figures presented were generated using *PyMOL* (v.1.5.0; Schrödinger).

## 3. Results and discussion   

### 3.1. Overall structure of the derCD23–Fc∊3-4 complex   

**Figure 1:**
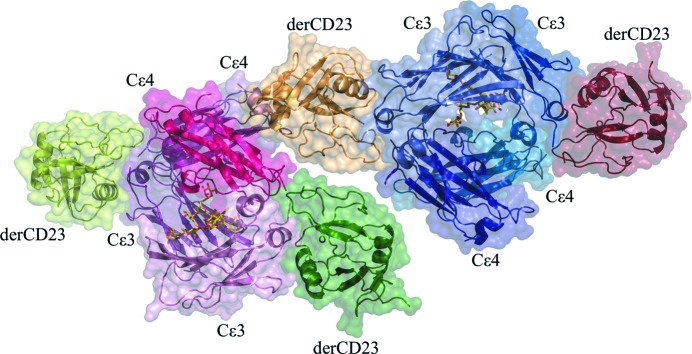
The asymmetric unit of the triclinic crystal form contains two independent copies of the derCD23–Fc∊3-4 complex. Two derCD23 head domains (displayed as C^α^ traces and surfaces coloured light and dark green) bind to one Fc∊3-4 dimer (coloured light and dark pink). The other two derCD23 domains (coloured light and dark brown) bind to the second Fc∊3-4 dimer (coloured light and dark blue). The carbohydrate is shown in all-atom representation (red and yellow, without surfaces).

The crystal structure reveals a complex consisting of one derCD23 head domain bound symmetrically to each IgE heavy chain between domains C∊3 and C∊4 (Fig. 1 ▶), as observed in the first crystal form of derCD23–Fc∊3-4 (Dhaliwal *et al.*, 2012 ▶). The earlier structure crystallized in a primitive orthorhombic lattice (space group *P*2_1_2_1_2_1_) with three complexes in the asymmetric unit, whereas the structure presented here crystallized in a triclinic lattice (*P*1) with two complexes in the asymmetric unit (Fig. 1 ▶) that are consequently subject to a different set of crystal-packing contacts. No Ca^2+^ ions were observed, as in the earlier structure, although in the presence of additional CaCl_2_ one Ca^2+^ ion binds to each derCD23 head in the same orthorhombic form; the structure of this Ca^2+^-bound complex has also been reported (Yuan *et al.*, 2013 ▶).

Comparisons of the individual C∊3 or C∊4 domains of Fc∊3-4 in the two crystal forms indicate virtually no structural differences, and the same is true for comparisons with the domains of unliganded Fc∊3-4 or IgE Fc (Wurzburg *et al.*, 2000 ▶; Wurzburg & Jardetzky, 2009 ▶; Wan *et al.*, 2002 ▶) or in complex with sFc∊RIα (Garman *et al.*, 2000 ▶; Holdom *et al.*, 2011 ▶). However, there are differences in their relative arrangement or quaternary structure that will be discussed in the following section.

**Figure 2:**
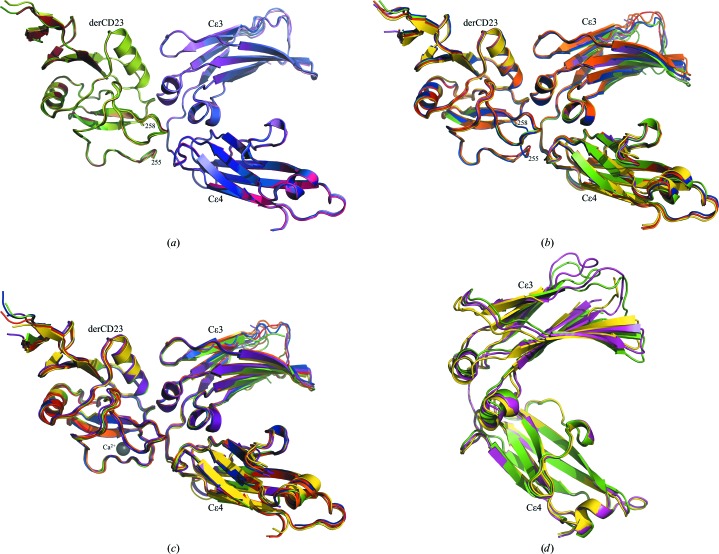
Comparison of the independent Fc∊3-4 and derCD23 molecules of the two crystal forms of the derCD23–Fc∊3-4 complex. (*a*) Superposition of the four Ca^2+^-free derCD23–Fc∊3-4 interactions in the triclinic crystal form. [The interacting chains (Fc∊34:CD23) *A*:*E*, *B*:*F*, *C*:*G* and *D*:*H* are coloured as in Fig. 1 ▶ and are superposed on the C∊4 and derCD23 domains.] (*b*) Superposition of the six Ca^2+^-free derCD23–Fc∊3-4 interactions in the orthorhombic crystal form (PDB entry 4ezm). [The interacting chains (Fc∊3-4:CD23) *A*:*G*, *B*:*H*, *C*:*I*, *D*:*J*, *E*:*K* and *F*:*L* are coloured red, orange, yellow, green, indigo and blue, respectively, and are superposed on the C∊4 and derCD23 domains.] (*c*) Superposition of the six Ca^2+^-bound derCD23–Fc∊3-4 interactions in the orthorhombic crystal form [PDB entry 4gko; same colouring scheme as in (*b*)]. (*d*) Superposition of Fc∊3-4 chains *C* (yellow) and *D* (green) of the Ca^2+^-free orthorhombic crystal form with chain *C* (pink) of the triclinic form (superposed on the C∊4 domains).

Comparison of the four derCD23 molecules in the present structure with the six in the earlier Ca^2+^-free structure (Dhaliwal *et al.*, 2012 ▶) shows that they are all virtually identical (Figs. 2 ▶ *a* and 2 ▶ *b*). This identity extends to the two highly mobile loop regions of derCD23, loop 1 (Leu226–Glu231) and loop 4 (Arg253–Glu257) (Hibbert *et al.*, 2005 ▶; Wurzburg *et al.*, 2006 ▶; Dhaliwal *et al.*, 2013 ▶), known to be critical for IgE binding (Bettler *et al.*, 1992 ▶; Yuan *et al.*, 2013 ▶), with residues Gly256 and Glu257 of loop 4 disordered as before. This observation of disorder in loop 4 in the absence of Ca^2+^ further supports our recent finding that this loop undergoes a conformational change with an accompanying ordering of the whole loop *only* upon the binding of Ca^2+^ to derCD23 (Fig. 2 ▶ *c*), a change that leads to further contact with IgE and explains the 30-fold enhanced affinity (Yuan *et al.*, 2013 ▶).

### 3.2. Quaternary domain structure of Fc∊3-4 in the complex   

Flexibility in the relative arrangement of the C∊3 and C∊4 domains, and variation in the C∊3–C∊4 interdomain angle, has been well documented in unliganded Fc∊3-4 (Wurzburg *et al.*, 2000 ▶; Wurzburg & Jardetzky, 2009 ▶), unliganded IgE Fc (Wan *et al.*, 2002 ▶), complexes of Fc∊3-4 or IgE Fc with sFc∊RIα (Garman *et al.*, 2000 ▶; Holdom *et al.*, 2011 ▶) and complexes of Fc∊3-4 with derCD23 (Dhaliwal *et al.*, 2012 ▶) (Yuan *et al.*, 2013 ▶). Comparing all of these structures, the interdomain angle varies over a range of ∼25°; Fc∊3-4 in complex with sFc∊RIα displays the most ‘open’ conformation, while the most ‘closed’ conformation observed to date is found in one of the Fc∊3-4 complexes in the earlier orthorhombic crystal form. The former is incapable of binding CD23 since the extreme ‘open’ angle restricts the available space at the interface of the two domains; the two spatially distinct receptor-binding sites located at either end of the C∊3 domains are thus allosterically linked through these relative domain motions (Dhaliwal *et al.*, 2012 ▶; Borthakur *et al.*, 2012 ▶).

Comparison between all of the CD23-bound Fc∊3-4 structures shows a more restricted but nevertheless still substantial range of interdomain angles. In the Ca^2+^-free orthorhombic crystal form the interdomain angle varies over a range of 7° between the six chains in the three independent complexes (Fig. 2 ▶ *b*). In the presence of Ca^2+^ this range (for the six chains) decreases to 3° (Fig. 2 ▶ *c*), but remarkably in the triclinic form reported here there is virtually no variation at all between the four independent chains (Fig. 2 ▶ *a*), which display an interdomain angle midway between the two extremes. This is illustrated in Fig. 2 ▶(*d*).

These different ranges of interdomain angles appear to be the result of crystal packing, since the number and distribution of such contacts differ considerably between the two crystal forms. In the triclinic crystals, all of the C∊3 domains have a high number of (atom-pair) crystal contacts, ranging from 50 to 75 over the four chains, whereas in the orthorhombic crystals a lower number of C∊3 contacts ranging from 18 to 50 is found. Although there is a smaller number of contacts, it is notable that chain *C* (the most open Fc∊3-4 conformation that binds to CD23; see Fig. 2 ▶ *d*) has 18 contacts, whereas chain *D* (the most closed Fc∊3-4 conformation seen to date; Fig. 2 ▶ *d*) has 50 C∊3 crystal contacts. (These contacts, presented in Supplementary Table S1^1^, are enumerated for the C∊3 domains only, since the C∊4 domain pair are closely associated with each other whereas the C∊3 domains are not, and thus the interdomain angle is most sensitive to the packing forces acting on C∊3.)

The fact that a range of C∊3–C∊4 interdomain angles is compatible with CD23 binding demonstrates that the plasticity seen at the IgE binding site in CD23 (Dhaliwal *et al.*, 2013 ▶) also extends to the IgE molecule. Furthermore, in solution, free from crystal-packing constraints, the IgE molecule presumably samples a range of angles at least as great as that reported in the crystal structures, although the C∊2 domains, if bent back and packing against one of the C∊3 domains (Wan *et al.*, 2002 ▶), may influence the degree of flexibility in the Fc∊3-4 region.

### 3.3. Additional interaction at the derCD23–Fc∊3-4 interface   

**Figure 3:**
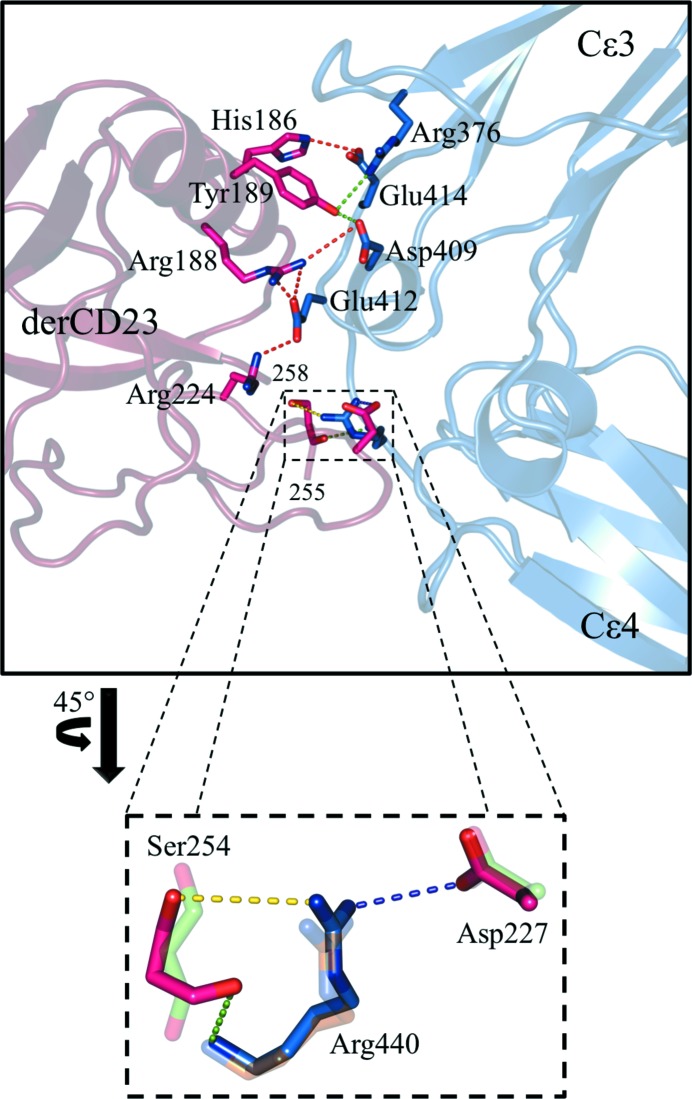
Salt bridges and hydrogen bonds at the derCD23–Fc∊3-4 interface of the triclinic crystal form. The four salt bridges shown in red, with additional hydrogen bonds shown in green, were seen in the orthorhombic crystal form. A further hydrogen bond present in three of four molecules is shown in yellow. Also depicted is an enlarged view of a region of the interface showing the additional Arg440–Asp227 salt bridge and hydrogen bond (in purple) found only in the triclinic crystal form. Lightly coloured residues are from the *A*:*G* interface of the orthorhombic crystal form (PDB entry 4ezm).

The derCD23–Fc∊3-4 interface (Fig. 3 ▶) is dominated by the four salt bridges and four hydrogen bonds that were observed in the orthorhombic crystal structure described previously (Dhaliwal *et al.*, 2012 ▶). However, one additional salt bridge and hydrogen bond, Arg440–Asp227, is observed in all four interfaces of the new triclinic crystal form and is not present in any of the six interfaces of the orthorhombic form. This hydrogen-bond distance ranges from 3.1 to 3.6 Å; the corresponding atoms in the orthorhombic crystal form are separated by distances of between 4.1 and 4.9 Å. To form the hydrogen bond, the side chain of Arg440 adopts a new orientation with its guanidinium moiety about 1.1 Å closer to the side chain of Asp258 in CD23. This hydrogen bond was also observed in the Ca^2+^-bound derCD23–Fc∊3-4 complex, where it forms one of six additional interface interactions as a result of the local structural rearrangement caused by binding of the cation (Yuan *et al.*, 2013 ▶). However, we now observe this additional salt bridge in the absence of Ca^2+^, a further example of the structural variation that occurs at this interface.

These results refine our understanding of the binding of IgE to CD23 and show that there is a remarkable degree of flexibility at the CD23–IgE interface not only in CD23 as reported earlier, but also in IgE. The different sets of crystal-packing forces acting on the complexes in the triclinic and orthorhombic forms, and the different C∊3–C∊4 interdomain angles in the four and six independent views of the interface in the two crystal forms, respectively, provide snapshots of the range of conformations that are compatible with CD23 binding. Understanding the extent of this plasticity may prove to be important in the design of either direct steric blocking agents or allosteric inhibitors of a protein–protein interaction that plays such a key role in the development of allergic disease.

## Supplementary Material

PDB reference: CD23–Fc∊3-4 complex, 4ki1

Supporting Information.. DOI: 10.1107/S2053230X14003355/tb5065sup1.pdf

## Figures and Tables

**Table 1:** Data-collection and refinement statistics Values in parentheses are for the outer resolution shell.

Data-processing statistics	
Wavelength (Å)	1.0332
Space group	*P*1
Unit-cell parameters (Å, °)	*a* = 48.79, *b* = 63.84, *c* = 163.89, α = 100.67, β = 90.13, γ = 103.49
No. of molecules in asymmetric unit	8
Solvent content (%)	57
Resolution range (Å)	80.4–3.20 (3.37–3.20)
No. of observations	114089
No. of unique reflections	30145
Average multiplicity	3.8 (3.9)
Completeness (%)	96.8 (98.4)
Wilson *B* factor (Å^2^)	61.5
〈*I*/σ(*I*)〉	3.4 (1.30)
*R*_p.i.m._ †	0.201 (0.547)
Refinement statistics	
Resolution range (Å)	80.4–3.20
Total No. of reflections	30144
No. of working reflections	28621
No. of test reflections	1523
*R*_xpct_ ‡	0.233
*R*_free_ §	0.266
No. of atoms	
Total	11158
Protein	10914
Carbohydrate	244
R.m.s. bond-length deviation (Å)	0.007
R.m.s. bond-angle deviation (°)	0.90
Mean *B* factor (Å^2^)	
Overall	79.3
Main chain	74.8
Side chain	83.8
Carbohydrate	99.5
R.m.s. backbone *B*-factor deviation¶	2.2
Ramachandran statistics†† (%)	
Favoured	95.4
Allowed	99.8
Outliers	0.2

† *R*_p.i.m._ (the precision-indicating merging *R* factor) = 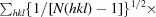 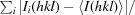 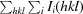 (Weiss, 2001 ▶).

‡ *R*_xpct_ = 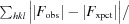 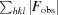, where |*F*_obs_| and |*F*_xpct_| are the observed structure-factor amplitude and the expectation of the model structure-factor amplitude, respectively (Blanc *et al.*, 2004 ▶).

§ *R*_free_ equals the *R*_xpct_ of the test set (5% of the data that were removed prior to refinement).

¶ R.m.s. deviation between *B* factors for bonded main-chain atoms.

†† As defined by *MolProbity* (Chen *et al.*, 2010 ▶).

## Footnotes

1 Supporting information has been deposited in the IUCr electronic archive (Reference: TB5065).
