# Supplementary material for: A range of C∊3–C∊4 interdomain angles in IgE Fc accommodate binding to its receptor CD23
Source: Acta Crystallogr F Struct Biol Commun. 2014 Feb 20;70(Pt 3):305–9. doi: 10.1107/S2053230X14003355 (PMC3944690; doi:10.1107/S2053230X14003355)
Supplement: Supplementary file 1 [file f-70-00305-sup1.pdf]

Supporting Information

Table S1. Cε3 crystal contacts.

(a) Ca^{2+}-free derCD23/Fcε3-4 triclinic crystal form contact analysis (<4.0A)

Chain	Total contacts
A	75
B	69
C	50
D	60

(b) Ca^{2+}-free derCD23/Fcε3-4 orthorhombic crystal form contact analysis (<4.0A)

Chain	Total contacts
A	36
B	44
C	18
D	50
E	24
F	18

(c) Ca^{2+}-bound derCD23/Fcε3-4 orthorhombic crystal form contact analysis (<4.0A)

Chain	Total contacts
A	46
B	24
C	10
D	56
E	13
F	13
